# Design, Development, and Evaluation of Treprostinil Embedded Adhesive Transdermal Patch

**DOI:** 10.3390/pharmaceutics15041226

**Published:** 2023-04-12

**Authors:** Ibrahim Alissa, Anroop B. Nair, Bandar Aldhubiab, Hiral Shah, Jigar Shah, Vivek Mewada, Rashed M. Almuqbil, Shery Jacob

**Affiliations:** 1 Department of Pharmaceutical Sciences, College of Clinical Pharmacy, King Faisal University, Al-Ahsa 31982, Saudi Arabia; 221401354@student.kfu.edu.sa (I.A.); baldhubiab@kfu.edu.sa (B.A.); ralmuqbil@kfu.edu.sa (R.M.A.); 2 Department of Pharmaceutics, Arihant School of Pharmacy & BRI, Adalaj, Gandhinagar 382421, India; vyashiral@yahoo.co.in; 3 Department of Pharmaceutics, Institute of Pharmacy, Nirma University, Ahmedabad 382481, India; jigsh12@gmail.com (J.S.); vmewada91@gmail.com (V.M.); 4 Department of Pharmaceutical Sciences, College of Pharmacy, Gulf Medical University, Ajman P.O. Box 4184, United Arab Emirates; sheryjacob6876@gmail.com

**Keywords:** treprostinil, transdermal, patch, optimization, pharmacokinetics, pulmonary arterial hypertension

## Abstract

Clinical application of treprostinil in pulmonary arterial hypertension is hampered by adverse effects caused by its high dosing frequency. The objective of this investigation was to Formulate an adhesive-type transdermal patch of treprostinil and evaluate it both in vitro and in vivo. A 3^2^-factorial design was utilized to optimize the selected independent variables (X_1_: drug amount, X_2_: enhancer concentration) on the response variables (Y_1_: drug release, Y_2_: transdermal flux). The optimized patch was evaluated for various pharmaceutical properties, skin irritation, and pharmacokinetics in rats. Optimization results signify considerable influence (*p* < 0.0001) of X_1_ on both Y_1_ and Y_2_, as compared to X_2_. The optimized patch possesses higher drug content (>95%), suitable surface morphology, and an absence of drug crystallization. FTIR analysis revealed compatibility of the drug with excipients, whereas DSC thermograms indicate that the drug exists as amorphous in the patch. The adhesive properties of the prepared patch confirm adequate adhesion and painless removal, while the skin irritation study confirms its safety. A steady drug release via Fickian diffusion and greater transdermal delivery (~23.26 µg/cm^2^/h) substantiate the potential of the optimized patch. Transdermal therapy resulted in higher treprostinil absorption (*p* < 0.0001) and relative bioavailability (237%) when compared to oral administration. Overall, the results indicate that the developed drug in the adhesive patch can effectively deliver treprostinil through the skin and could be a promising treatment option for pulmonary arterial hypertension.

## 1. Introduction

Pulmonary arterial hypertension (PAH) is a rare, extremely complex, and progressive condition that is eventually fatal and can cause early mortality [1]. This uncommon condition is characterized by unfavorable arterial tree remodeling that causes increased vascular resistance, an increase in right ventricular afterload, and eventually results in heart failure [2]. Early symptoms are nonspecific and typically include dyspnea, angina, exertion, and fatigue. Nonspecific clinical symptoms and pathological ignorance result in a poor prognosis, which may be related to the delay in diagnosis/misdiagnosis and treatment initiation. There is currently no known cure for PAH despite the fact that it can be treated with various therapies [3]. Indeed, the epidemiology and management of this devastating disease have seen a significant change in the last few decades. Several drug molecules from various drug classes are currently approved for PAH treatment, including ambrisentan, bosentan, epoprostenol, riociguat, sildenafil, tadalafil, and treprostinil, which target different biological pathways [4]. Current pharmacological therapies target the endothelin, nitric oxide, or prostacyclin pathways in an effort to delay the progression of the disease and lessen symptoms. Among these, the drugs that target the prostacyclin pathway have indeed been demonstrated to be beneficial for patients as they possess anti-proliferative, anti-inflammatory, and vasodilatative actions [5]. 

Treprostinil is a prostacyclin (PGI_2_) analog that reduces pulmonary arterial pressure by effectively dilating both systemic as well as pulmonary arteries [5]. This drug is available in three separate dosage forms, including injection, inhalation, and oral tablet [6,7]. However, the available dosage forms do possess specific complications apart from the intolerable adverse effects. For instance, the inhalation therapy of treprostinil could cause both local and systemic side effects [8]. Similarly, parenteral therapy primarily causes local adverse effects like discomfort and swelling where injections are made, which frequently results in limiting the dose escalation or therapy discontinuation. The cause of such serious side effects is primarily due to higher treprostinil levels in the blood or at the target site (lungs) because of continuous infusion or multiple dosing [9]. The high dosing frequency of treprostinil is directly linked to its short biological half-life (3–4 h). Depending on the route of administration, treprostinil may need to be given as often as every 2 h to maintain therapeutic levels [10]. All these above-mentioned issues have limited the clinical use of treprostinil [5]. Thus, developing a delivery system that can offer a sustained release of treprostinil and maintain the steady drug level for a long period with less dosing frequency could be of clinical benefit. A few studies have been conducted to reduce the side effects as well as to improve the efficiency of this drug by preparing prodrug or developing extended-release tablets or nanoparticles, which yielded moderate improvement in clinical outcomes [8,11,12]. 

Transdermal therapy is a noninvasive approach that allows self-administration by the patient and is considered one of the most promising approaches in the last few decades [13]. These systems are primarily meant to deliver the drug through the skin membrane and enter the systemic circulation, wherein it can provide continuous and consistent blood levels for a prolonged period. Transdermal therapy conquers the difficulties of the oral route and has great potential to be an effective route, while the plasma profile of the transdermal system is comparable to intravenous infusion. In general, the first-pass metabolism, toxicity, and variation in the plasma drug concentration are controlled in transdermal therapy. The stratum corneum has multiple layers of dead keratin-rich cells and is a major barrier to the percutaneous absorption of drugs. Drug factors, including molecular mass, melting point, log P, pKa, and dose, influence the passage of molecules into and through the skin [14]. Transdermal therapy has been effective in delivering a wide range of therapeutic agents, particularly in treating cardiovascular and nervous system disorders, managing pain, and providing hormone therapy [15]. Most of the commercially available transdermal systems belong to the drug in adhesive type, owing to their potency to offer controlled release/permeation of the drug, long skin residence time, ease of formulation, as well as better patient compliance [16]. This thin polymeric membrane-based formulation can be easily fabricated, and the release of therapeutic molecules could be managed by altering the polymer/adhesive components. Pressure-sensitive adhesives or polymeric adhesives are typically used in such systems and depend on the physical and chemical characteristics of the drug [17]. Chemical enhancers are generally incorporated in these formulations to improve the percutaneous absorption of drug molecules with low permeability [18,19]. Literature indicates that several investigations were conducted in the last decade using the drug in the adhesive system to improve the efficacy of various drug molecules [16,20,21,22,23]. Given the importance of transdermal therapy and the issues with treprostinil, we hypothesized that developing a transdermal delivery system would be ideal for treating PAH. Moreover, the drug properties of treprostinil, like low MW (~390 Da), melting point (~122 °C), dose (<5 mg), and short half-life (~4 h), favor its suitability for transdermal delivery. Thus, the aim of this research was to design and formulate an adhesive transdermal delivery system of treprostinil and evaluate its efficacy in rats. Optimization of the formulation was carried out by a 3^2^-full factorial design. The effect of formulation parameters on the outcome of the product as well as the therapeutic effect, was assessed. 

## 2. Materials and Methods

### 2.1. Materials

Treprostinil was obtained as a gift sample from Emcure Pharmaceuticals Ltd., Ahmedabad, India. DuroTak 87-2287, DuroTak 87-4098, and DuroTak 87-2852 (National Starch and Chemical Company, Bridgewater, NJ, USA), ScotchPakTM 9744 (3M, St. Paul, MN, USA), ethyl acetate (Sigma Aldrich, Darmstadt, Germany), propylene glycol (Sigma Aldrich, Darmstadt, Germany) were purchased commercially. The other chemicals and reagents purchased were used as received and were of reagent grade or above. 

### 2.2. Estimation of Treprostinil by High-Performance Liquid Chromatography (HPLC)

Detection of treprostinil in samples was conducted using an HPLC system (Agilent Technologies, Waldbronn, Germany) equipped with a Kromasil C18 column (4.6 × 250 mm, 5 μm). A solvent combination of acetonitrile and water (40:60 *v*/*v*) with a pH of 4 adjusted using trichloroacetic acid was used as the mobile phase. The analytical condition for separation of the drug was; flow rate (1.5 mL/min), column temperature (25 °C), run time (15 min), internal standard (kanamycin sulfate), injection volume (50 μL), and detection wavelength (220 nm). Specimens collected were filtered using a hydrophilic polyvinylidene difluoride membrane with a pore size of 0.45 µm and sonicated for 5 min before injecting in the HPLC system.

### 2.3. Preparation of Drug in Adhesive Transdermal Patch

The solvent evaporation method described before was used to formulate the treprostinil-containing drug in adhesive patches [24]. Briefly, a specific amount of treprostinil (0.3 or 0.5, or 0.7% *w*/*w*) was dissolved in ethyl acetate, and to this solution, the required quantity of permeation enhancer was incorporated and mixed thoroughly to get uniform dispersion. To this mixture, the pressure-sensitive adhesive was added and vigorously stirred for 1 h. A custom-made film applicator coater was used to apply the resultant solution on the release liner’s surface to get a thickness of 2000 µm. The drug-pressure sensitive adhesive layer was then dried at 50 °C for 15 min in a laboratory oven and then for 15 min at ambient temperature. The backing film was then fixed over the drug-adhesive layer using a hand roller. Similarly, the control patch was prepared without permeation enhancers. Finally, the proper sizes of the patch were cut, covered in aluminum foil, and stored at room temperature.

### 2.4. Screening of Transdermal Patch Components 

Identification of adhesive, release liner, backing membrane, and chemical enhancers for the proposed transdermal patch was performed. Three acrylate adhesives (DuroTak 87-2287, DuroTak 87-4098, and DuroTak 87-2852) were used to prepare patches, and their effect on permeation was assessed. The other components like treprostinil (0.5%), ScotchPak™ 9744 (release liner), ScotchPak™ 9723 (backing membrane), and propylene glycol (5%, permeation enhancer) were fixed. Similarly, three commonly used release liners, namely ScotchPak™ 9744, ScotchPak™ 1022, and ScotchPak™ 9741, were evaluated by keeping other components like treprostinil (0.5%), DuroTak 87-2287 (adhesive), ScotchPak™ 9723 (backing membrane), and propylene glycol (5%, permeation enhancer) were fixed. Properties like peeling and adhesiveness were checked for selecting release liners. The effect of the backing membrane was tested using CoTran™ 9707, CoTran™ 9722, and ScotchPak™ 9723. Treprostinil (0.5%), DuroTak 87-2287 (adhesive), ScotchPak™ 9744 (release liner), and propylene glycol (5%, permeation enhancer) were fixed, and the effect of drug permeation was assessed. The impact of chemical enhancers on drug permeability was assessed using 5% of oleic acid, propylene glycol, Tween 80, methanol, and azone. The other components, like treprostinil (0.5%), DuroTak 87-2287 (adhesive), ScotchPak™ 9744 (release liner), and ScotchPak™ 9723 (backing membrane), were fixed.

### 2.5. Preliminary Trial Batches of Transdermal Patch

The effect of drug level (0.1–1% *w*/*w*) and concentration of penetration enhancer (propylene glycol, 1–10% *w*/*w*) on treprostinil permeation was assessed by formulating 20 formulations (F1-F20). The concentration of the drug and permeation enhancers are described in Table S1. The other formulation components, like DuroTak 87-2287 (adhesive), ScotchPak™ 9744 (release liner), and ScotchPak™ 9723 (backing membrane), were fixed. 

### 2.6. Factorial Design 

The factorial design allows for the identification and evaluation of the relative importance of the various components that influence a particular process. As a result, it provides a way to distinguish between crucial and irrelevant factors [25]. In addition, this method identifies any possible interactions between the selected elements. Therefore, choosing the parameters and responses are both necessary for the creation of a factorial design. In this study, the data were analyzed utilizing Design-Expert (version 13) software to determine the best treprostinil adhesive patch using a two-factor, three-level factorial design (3^2^). Following the preliminary investigation, a thorough analysis of the two formulation variables was carried out using the design-of-experiments approach to determine the ideal combination of the independent variable for the preparation of patches with the targeted drug release rate and permeation flux. The drug quantity (%) (X_1_) and enhancer concentration (%) (X_2_) were chosen as the independent variables (factors), while the drug release (%) (Y_1_) and transdermal flux (µg/cm^2^/h) (Y_2_) were the response variables (dependent). The factors, their levels chosen, and the dependent variables used in the factorial design are summarized in Table 1 and Table 2. Accordingly, nine batches (F1–F9) were prepared and evaluated for dependent variables (Y_1_ and Y_2_). The optimized batch was prepared using values of independent variables, X_1_ − 0.890 (0.678% *w*/*w*) and X_2_ − 0.368 (5.736% *w*/*w*), selected from the overlay graph according to the point prediction method.

### 2.7. Drug Release

Release of treprostinil from prepared patches was assessed using Franz diffusion setup (Logan Instruments Ltd., Somerset, NJ, USA). A dialysis membrane with a specific aperture size of 2.4 nm (Himedia, Mumbai, India) was placed between the chambers, which acted as a release barrier [26]. The receiver media (5 mL) used was PBS (pH = 7.4) containing Tween 80 (10%) and was agitated (50 rpm) using a small bead. The experiment was performed by maintaining the temperature (32 ± 1 °C). The area present for drug diffusion was 0.74 cm^2^. The patch containing treprostinil 1 mg/cm^2^) was placed over the skin and pressed to have enough contact with the membrane. The donor chamber was then covered with Parafilm. Samples (2 mL) were filtered and estimated by HPLC. The total drug release (%) versus time was evaluated using various kinetic models to identify the possible mechanisms of treprostinil release from the optimized patch [27,28]. 

### 2.8. Characterization of Transdermal Patch

#### 2.8.1. Drug Content

The treprostinil content in the prepared transdermal patches was measured in a 1 cm^2^ area of the patch. The patch was immersed in the mobile phase (10 mL) and allowed to sonicate for 60 min. The mobile phase was further centrifuged (12,000 rpm for 5 min), filtered, and analyzed the supernatant solution using HPLC.

#### 2.8.2. Microscopic Observations

The morphology of the patches was examined under an optical microscope (CX21iLED, Olympus, Hamburg, Germany). The section of the prepared adhesive patch was kept on a glass plate and was viewed under a microscope with a 40× objective and 10× ocular lens [20]. Scanning electron microscopy (SEM, ULTRA 55, Carl Zeiss, Jena, Germany) was employed to investigate the surface analysis of the patches. The patches were analyzed using an Ultra-55 Carl Zeiss field emission. The patches were mounted onto 12 mm aluminum pin stubs with double-sided adhesive tapes. The stubs were then coated with gold using a sputter coater under high vacuum and voltage. The sample images were captured using an electron beam (5 kV to 15 kV) at magnification from 500× to 10k×.

#### 2.8.3. Fourier Transform Infrared Spectroscopy (FTIR) Study

The spectra of treprostinil, the blank patch, and the optimized patch were evaluated by employing an FTIR spectrometer (6100, Jasco, Tokyo, Japan). Chloroform was used to dissolve the samples and placed (0.1 mL) in the liquid sample holder. The samples were scanned (14 scans) with a resolution set at 16 cm^−1,^ and the spectra were monitored between 400 cm^−1^ to 4000 cm^−1^ region.

#### 2.8.4. Differential Scanning Calorimetry (DSC)

The thermodynamic characteristics of treprostinil, blank patch, and optimized drug in the adhesive patch were evaluated by DSC (Hitachi DSC 7020, Tokyo, Japan). A piece of patch or a small amount of drug was kept in aluminum crimped pans and sealed, with an empty pan serving as the standard reference [29]. A nitrogen atmosphere with a temperature range of 30–300 °C was used to record the thermograms, which were heated at a rate of 10 °C/min.

#### 2.8.5. Coat Weight

The optimized patch with an area of 0.28 cm^2^ was cut using a biopsy punch and then minus the weight of both the backing membrane and release liner to determine the coat weight as described elsewhere [21]. 

#### 2.8.6. Adhesion Testing 

The adhesive nature of the prepared drug in the adhesive patch was used to determine the peel adhesion and tackiness using a texture analyzer by following standard procedures. A peel adhesion test was carried out with a 5 kg load cell. The optimized transdermal patch was cut into strips of appropriate size (length-7 inches and width 2 inches) and was attached to the metal surface, and one end of the patch was attached to the movable probe. The release liner was taken off (removed) prior to the test. The probe was pulled up at 180° at a speed of 30 mm/min, and the peak load was measured to complete the peel of the patch from the metal surface [30]. Tackiness was performed using a texture analyzer equipped with a 5-kg cell. An optimized transdermal patch with a 2 inch × 2 inch size was placed on the lower probe. The release liner was taken off prior to the test. The texture analyzer’s cylindrical 30-mm-diameter alum probe was lowered at a pace of 20 mm/min with a 5-kg load cell attached to press patches with a 100-g force [30]. The highest peak load necessary to separate the patch was recorded when the probe was moved up at a rate of 20 mm/min.

### 2.9. Skin Irritation

Rats were divided into three groups, with each group having six animals. The hairs on the dorsal side were removed, and the procedure was carried out every day for three days in a row on a 1.5 cm^2^ region. Group 1 received a typical skin irritant (2.5% sodium dodecyl sulfate), Group 2 received a transdermal patch containing a placebo, and Group 3 received an optimized adhesive patch containing treprostinil. According to the Draize dermal scoring guidelines, the region where the product was administered was evaluated for erythema and edema and given a score [31].

### 2.10. Ex Vivo and In Vivo Studies

Wistar rat skin membrane was used in the ex vivo permeation studies using Franz permeation setup (Logan Instruments Ltd., Somerset, NJ, USA). Rats were sacrificed, and the skin layer was collected after removing the hairs. The experimental setup was similar to the procedure described in drug release (Section 2.7). The skin membrane was placed between both chambers while the stratum corneum faced the donor. A circular patch containing treprostinil (1 mg/cm^2^) was placed over the skin and pressed to have enough contact with the skin. The patch without enhancer was used as a control. Samples (2 mL) were filtered and estimated by HPLC.

In vivo, the study was carried out in male Wistar rats (200–250 g), which were kept in an animal room with access to standard feed and water. Animals were treated in strict accordance with the Institutional Animal Ethics Committee’s ethical regulations (KFU-REC-2021-NOV-EA000155, dated; 9 November 2021). Twelve animals were used for the investigation and were distributed into two separate groups. The group 1 rats were anesthetized by injecting ketamine (40 mg/kg) + xylazine (5 mg/kg) intraperitoneal, and the dorsal hair was trimmed. The optimized patch (area of 1 cm^2^) having 1 mg/rat of treprostinil was placed on the skin surface, and applied slight pressure to ensure adequate adhesion. In the control group, the drug suspension (1 mg/rat) was administered peroral using intra-gastric gavage. Blood samples (200 µL) were collected in a heparinized tube from the tail vein at various time intervals for 24 h. Dextrose (250 µL) was injected intraperitoneally after every blood sampling to avoid a reduction in the volume of the central compartment. Samples were protein precipitated and injected into HPLC. The pharmacokinetic parameters, such as the maximal plasma drug concentration (C_max_), the time to the maximal drug concentration (T_max_), and the area under the plasma concentration-time curve from time zero to infinity (AUC_0-α_), were calculated. 

### 2.11. Statistical Analysis

The optimization data were analyzed using Design Expert software (Design Expert v.13, State-Ease, Inc., Minneapolis, MN, USA) by ANOVA to check the significance between different effects. Comparison of other effects was performed by one-way ANOVA or Student *t*-test using GraphPad Prism software (version 6, GraphPad, San Diego, CA, USA). The significance level was chosen at *p* < 0.05. The values presented are average and standard deviation.

## 3. Results and Discussion

An HPLC analytical method was developed for the estimation of treprostinil in various samples. The chromatogram peak of the drug observed at 11.2 min was used as the criterion to quantify the treprostinil. The calibration curve prepared in the concentration range of 20–1000 ng/mL showed an excellent regression coefficient (r^2^ = 0.9981).

### 3.1. Screening of Transdermal Patch Components

Formulation components like adhesives, release liner, backing film, drug loading, etc., generally influences the efficacy of transdermal patches [22,32]. The physicochemical properties of adhesives, like the functional groups, can impact drug permeation. Therefore, three acrylic adhesives (DuroTak 87-2287, DuroTak 87-4098, and DuroTak 87-2852) with different functional groups were selected and evaluated for permeation. Typically, the selected adhesives differ in their functional groups; DuroTak 87-2287 contains hydroxyl groups, DuroTak 87-4098 has no functional group, while DuroTak 87-2852 has carboxylic groups. The permeation rate (25.35 µg/cm^2^/h) was found to be higher with DuroTak 87-2287, probably due to the chemical nature of the adhesive [33]. The drug permeation was significantly low (*p* < 0.0001) with DuroTak 87-4098 (15.09 µg/cm^2^/h) and DuroTak 87-2852 (9.78 µg/cm^2^/h). Based on these results, DuroTak 87-2287 was selected. 

The selection of release liners was made by measuring their ability to remove easily from dried formulation without leaving any residue. The results showed ScotchPak™ 9744 could easily peel off from the adhesive and have no formulation residue, as compared to other release liners tested. Hence, ScotchPak™ 9744 could be suitable for the development of the current formulation. In the next phase, the effect of backing membranes on permeation was assessed. A moderate influence of the backing membrane on the transdermal flux of treprostinil was noticed, and the flux was relatively higher with ScotchPak™ 9723. Permeation enhancers are vital components in transdermal formulations as they have an important role in improving the transdermal flux of drugs [34,35]. In addition, these agents are capable of avoiding phase separation, improving the drug’s solubility in adhesives, and thereby enabling higher drug concentration in the patch [21]. Therefore, in the final phase, the effect of five well-known chemical enhancers (at 5% *w*/*w*) in enhancing the transdermal flux of treprostinil was assessed. Indeed, a considerable difference in the drug permeation was noticed with various chemical agents tested. A greater flux (~25.19 µg/cm^2^/h, *p* < 0.005) was noticed with propylene glycol when compared with other chemicals tested. The enhancement in flux decreases as; Tween 80 > oleic acid > methanol > azone. The potential of propylene glycol to improve the skin permeation of various drug molecules is described in the literature. The possible mechanisms for its effect are mainly due to its potential to enhance the drug partition into the skin, increase lipid fluidity, and hence permeation [36]. In addition, the patches prepared were soft and flexible and had a thickness in the range of 1300–1600 µm.

### 3.2. Preliminary Trial Batches of Transdermal Patch

The preliminary data (Table S2) suggests that the increase in the drug level improves the transdermal flux of treprostinil to a certain level (0.5% *w*/*w*) and then plateaus indicating saturation. An increase in the drug level above 0.5% *w*/*w* did not show any significant improvement in the drug permeation. Similarly, the increase in the chemical enhancer concentration also showed improvement in the flux, and this was prominent when the concentration was 1–5% *w*/*w*. Based on the preliminary data, a 3^2^ optimization design was employed to study the effect of these two components on the overall treprostinil transdermal flux in more detail.

### 3.3. 3^2^ Full Factorial Design

To determine the impact of factors on each dependent variable, the received responses were fitted to multiple linear regression analysis models. The representation of the observed polynomial equation is;

Yi = b0 + b_1_X_1_ + b_2_X_2_ + b_12_X_1_X_2_ + b_11_X_1_^2^ + b_22_X_2_^2^

This equation shows the main factors (X_1_ and X_2_), interaction factors (X_1_X_2_), and polynomial factors (X_1_^1^ and X_2_^2^). The b0 represents the overall mean of total runs, and b_1_, b_2_, b_12_, b_11,_ and b_22_ are the projected coefficients for factors X_1_ and X_2_. The polynomial equation was used to conclude after evaluating the coefficient magnitudes and corresponding mathematical symbols, which may be positive or negative. In general, significant model terms are indicated by the greater coefficient, whilst low values will be seen in non-significant models. A positive sign signifies that increasing the level of one variable in the polynomial equation will result in an improvement in that specific response and vice versa. In general, an ANOVA test was carried out to examine the level of significance of both models as well as their terms. The terms are considered significant when the observed *p*-value is less than 0.05. The Design-Expert program was used to prepare the 2D-contour plots, as well as 3D-response surface plots to illustrate and better comprehend the connections between the independent and dependent variables. Additionally, the correlation plots made between the real and expected values served as further evidence of the model’s validity. The excellent match is indicated by the high R^2^ values (>0.9990) between the real and expected values for each response. Based on the software’s point prediction method, the best formulation was chosen, and the formulation parameters were optimized using the quadratic model. 

Based on the software recommendations, nine transdermal patches were prepared by varying two components (drug concentration and enhancer concentration) at three different levels and tested for in vitro drug release and transdermal permeation. The observed responses (drug release and transdermal flux) of nine prepared formulations using factorial design are summarized in Table 3. The target criteria of dependent variables like drug release (maximum) and transdermal flux (maximum) were set to achieve by altering the independent variables of the transdermal patch. The results of data analysis to determine the variables influencing statistically significant responses are displayed in Tables S3 and S4.

#### 3.3.1. Effect on Drug Release

The release of drugs from the adhesive patches is a primary requisite for its diffusion through the skin membrane and exerts a systemic effect. The rate and extent of drug release are typically regulated by both drug dissolution and its diffusion coefficient in the polymer matrix [37]. Thus, the influence of the amount of drug as well as an enhancer in the patch on drug release was assessed by a design-of-experiments approach. 

The statistical analysis data observed indicate that the model is significant (*p* < 0.0001), where both independent variables affect drug release. The observed drug release of prepared formulations varied widely and was in the range of ~45–86% (Table 3), indicating the factors affect the response significantly (*p* < 0.0005). The polynomial equation for response Y_1_ is;

Y_1_ (drug release) = +64.79 + 15.38 X_1_ + 4.88 X_2_ − 0.2750 X_1_X_2_ + 0.4550 X_1_^2^ + 0.5750 X_2_^2^

**Figure 1:**
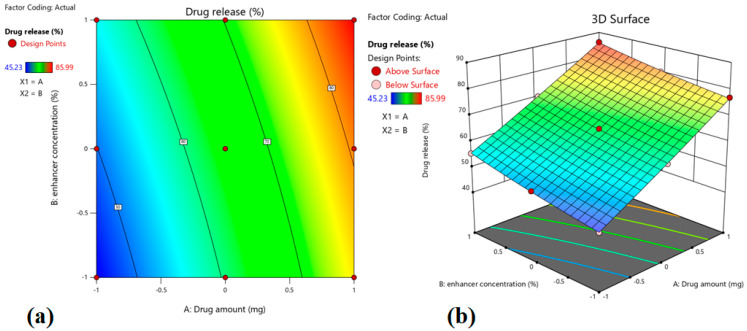
Contour plot (**a**) and 3D surface plot (**b**) show the effect of drug amount and enhancer concentration on drug release.

**Figure 2:**
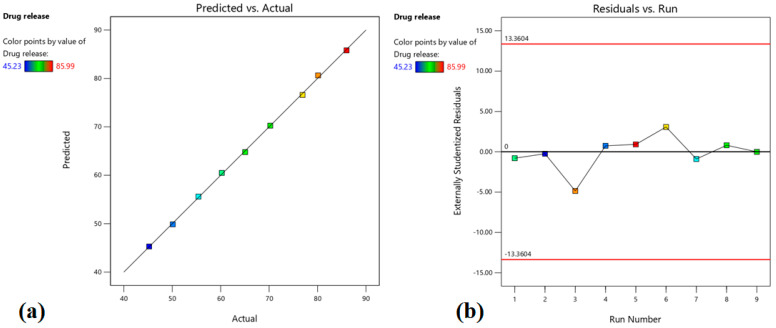
Linear correlation plot of predicted vs. actual (**a**) and related residual plot (**b**) on drug release.

The positive value in the above equation indicates that drug release is proportional to both the independent variables (X_1_ and X_2_). The higher value of X_1_ (+15.38) signifies that the drug amount has a greater influence (*p* < 0.0001) on the drug release (%), which increases with the rise in the drug amount (0.3–0.7% *w*/*w*) in prepared patches. This fact can be explained by comparing batches 2 and 6, where increasing the drug amount from −1 (0.3%) to 1 (0.7%), there is an increase in drug release from 45.23% to 76.91% (Table 3). Although the value for X_2_ was lower (+4.88) than X_1_, the enhancer concentration also showed significant influence (*p* = 0.0001) on the drug release, which can be explained by comparing batches 2 and 7, where increasing the enhancer concentration from −1 (3%) to 1 (7%), there is an increase in drug release from 45.23% to 55.41%. The contour, as well as the 3D surface plot showing the effect of drug amount and enhancer concentration on drug release, were presented in Figure 1. By analyzing Figure 1, it is evident that X_1_ has a greater impact on drug release compared to X_2_. Figure 2a quantitatively compares the resultant actual values for drug release with those of the predicted values, while Figure 2b represents the corresponding residual plots for drug release. The predicted (theoretical) values and the actual (experimental) values were in reasonably good agreement. These results confirmed the validity of the design model developed.

**Figure 3:**
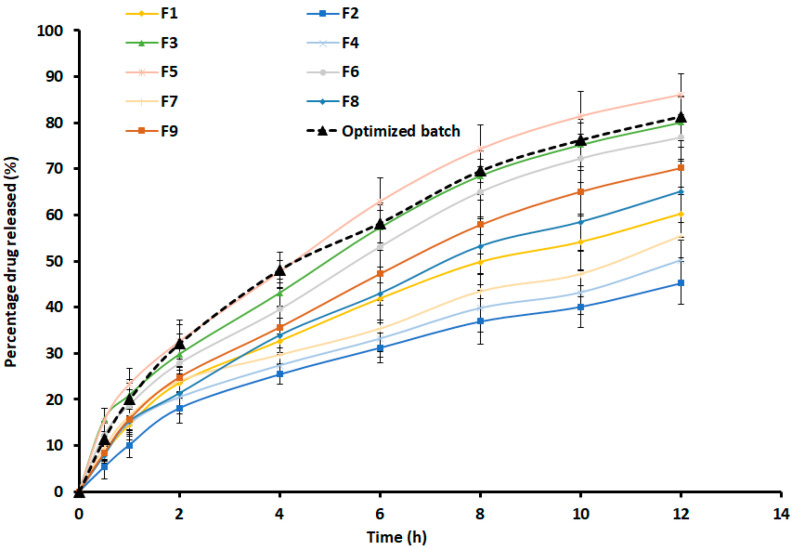
Release of treprostinil from design batches (F1–F9) and optimized transdermal patch (n = 6).

Figure 3 presents the release profiles of optimized and designed batches. The profiles show the cumulative percentage of drug release, which varies across patches tested and is primarily influenced by drug amount, as observed in the polynomial equation. In batches F2, F4, and F7, where the drug level was 0.3% *w*/*w*, the cumulative amount of treprostinil release was relatively low (45–55%). However, the drug release was moderate (60–70%) in batches F1, F8, and F9 when the drug level was increased to 0.5% *w*/*w*. Further, increase in drug amount in patches to 0.7% *w*/*w*, and the release also increased (76–86%) as noticed in batches F3, F5, and F6. These profiles clearly demonstrated the effect of treprostinil concentration on its release from design batches. The possible explanation for this effect could be related to the ratio of polymer to the drug in the patches. In the case of 0.3% *w*/*w* batches, the amount of drug was low; hence the fraction of polymer to drug ratio was high. However, when the amount of drug in the patches increased, the fraction decreased. Therefore, a greater amount of polymer would have produced a dense polymeric network when exposed to water, reducing drug diffusion and release, as described in the literature [38]. Similarly, the variation in chemical enhancer concentration in prepared patches showed a considerable effect on drug release. The effect can be easily evidenced by comparing enhancer concentrations of 3% *w*/*w* in batches F1, F2, and F6 (45–76%) with batches F5, F7, and F9 (55–86%) having higher enhancer concentrations of 7% *w*/*w*. This finding aligns with previous studies that have shown how higher concentrations of enhancers lead to an increase in drug release [38,39]. The possible reasons for the enhancement in drug release by the hydrophilic propylene glycol (enhancer) to form hydrophilic pores inside adhesive and support water uptake are generally observed when used in matrix patches [38,39].

The release of treprostinil from the selected patch was presented in Figure 3, which showed a steady increase in treprostinil release with ~32% (2 h), ~48% (4 h), ~58% (6 h), and ~81 (12 h). Such a release profile is ideal for transdermal therapy as the drug will be steadily released on the skin surface for constant diffusion through the skin. However, it is essential to understand the diffusion mechanism of drug molecules in the adhesive matrix while designing transdermal patches [37]. Hence, the release mechanism of the optimized patch was assessed by fitting the data in different mathematical models, and the data are presented in Table S5. The highest r^2^ value (0.9989) with low SSR (7.7355) and Fischer ratio (1.1051) was observed with the Weibull model, indicating that the release of treprostinil from the optimized patch followed Weibull kinetics. The estimated slope value of 0.6635 was less than 0.75, which suggests the drug release was Fickian diffusion [40], which is a material transport process generally noticed in adhesive matrixes [37].

#### 3.3.2. Effect on Transdermal Flux

The efficacy of a transdermal delivery system relies on its ability to provide optimal drug release for an extended period, along with adequate percutaneous absorption [16]. Indeed, the transport of therapeutic actives through the skin membrane is affected by both the physicochemical characteristics of the diffusant as well as the anatomy and physiology of the membrane [41,42]. The role of chemical agents in augmenting the transport of drugs through the formidable skin barrier is established. In addition, the mechanisms of action of these agents are also well-understood [43]. Hence, the influence of the amount of drug as well as an enhancer in the patch, on the transdermal flux of treprostinil was assessed by the design of experiments. The statistical analysis data observed indicate that the model is significant (*p* = 0.0004 value), where both independent variables affect the transdermal flux. The observed transdermal flux of prepared formulations varied widely and was in the range of ~10–27 µg/cm^2^/h (Table 3), indicating the factors affect the response significantly (*p* < 0.0001). The polynomial equation for response Y_2_ is Y_2_ (transdermal flux) = +16.46 + 5.21 X_1_ + 3.05 X_2_ + 0.5500 X_1_X_2_ + 0.9867 X_1_^2^ + 0.7067 X_2_^2^

The positive value in the above equation indicates that transdermal flux is proportional to both the independent variables X_1_ and X_2_. Meanwhile, one should bear in mind that the observed polynomial equation for transdermal flux observed was in rat skin, and the results may not necessarily translate directly to humans. However, the polynomial equations observed in rats can provide relevant information to construct a well-targeted DOE for an in vivo study in humans [38,44,45]. The higher values of X_1_ (+5.21) and X_2_ (+3.05) observed in this investigation signify that the drug amount, as well as the concentration of enhancer, have considerable influence on the transdermal flux (*p* < 0.0001 and *p* = 0.0004, respectively), which increases with the rise in the drug amount (0.3–0.7% *w*/*w*) and enhancer (3–7% *w*/*w*) in patches. By comparing batches 4 and 5, it can be interpreted that increasing the drug amount from −1 (0.3%) to 1 (0.7%) and raising the enhancer concentration from 0 (5%) to 1 (7%) results in the enhancement of transdermal flux from 12.12 µg/cm^2^/h to 26.98 µg/cm^2^/h (Table 3). 

**Figure 4:**
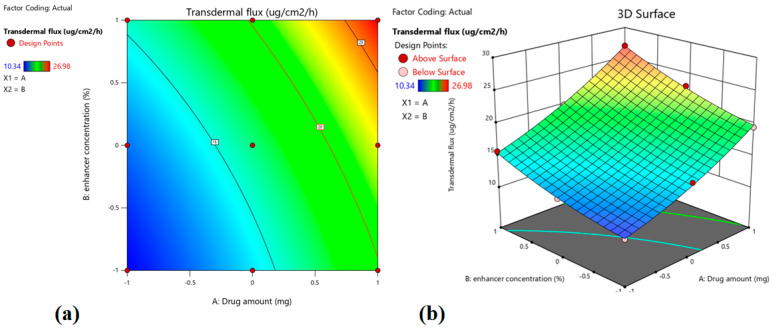
Contour plot (**a**) and 3D surface plot (**b**) showing the effect of drug amount and enhancer concentration on transdermal flux.

**Figure 5:**
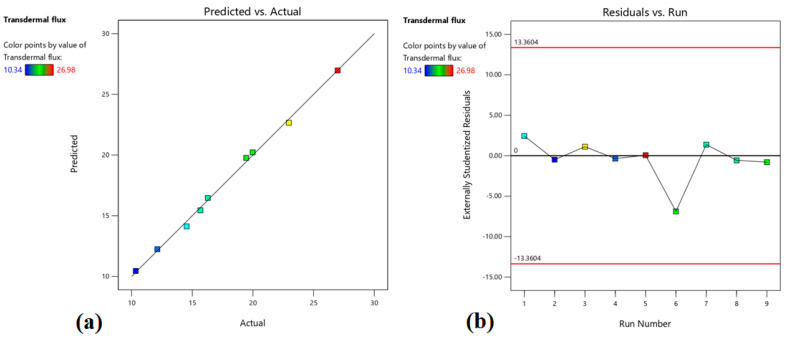
Linear correlation plot of predicted vs. actual (**a**) and related residual plot (**b**) on transdermal flux.

The contour plot, as well as the 3D surface plot showing the impact of drug level and enhancer concentration on transdermal flux, are shown in Figure 4. It is noticed that both independent factors have a positive impact on transdermal flux. Figure 5a compares the transdermal flux (quantitatively) of the resultant actual values with those of the predicted ones, while Figure 5b represents the corresponding residual plots for transdermal flux. The predicted (theoretical) values and the actual (experimental) values were in reasonably good agreement. These results confirmed the validity of the design model developed.

**Figure 6:**
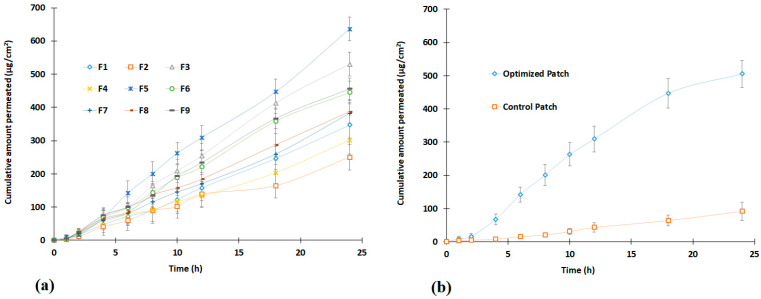
Permeation profiles of treprostinil from design batches (F1–F9) (**a**) and optimized transdermal patch (**b**) (n = 6). The control patch contains the same amount of drug without enhancers.

The permeation of treprostinil through the skin from design batches, optimized batches, and control is presented in Figure 6. The amount of drug permeated from design batches varied among the tested patches and was influenced by both drug content and enhancer concentrations studied, as noticed in Figure 6. A direct correlation between the treprostinil amount in the patches and the transdermal flux can be seen in Figure 6. For instance, the effect can be easily evidenced by comparison of profiles of batches F1 (0.5% *w*/*w*), F2 (0.3% *w*/*w*), and F6 (0.7% *w*/*w*) with different drug levels though the enhancer concentration is the same (3% *w*/*w*). Moreover, the cumulative amount of drug permeated from these batches at the end of 24 h was found to be statistically significant (*p* < 0.0001). The enhancement in treprostinil permeation with an increase in drug amount in prepared patches could be directly linked to their thermodynamic activity. In general, the transport of drugs through the skin is a passive diffusion process and is mainly influenced by the concentration gradient. Hence higher drug levels could yield greater flux, as observed in several studies [20,24,46]. 

The influence of chemical enhancers on the percutaneous absorption of treprostinil could also be evidenced in Figure 6. For instance, a comparison of batches F2, F4, and F7 with low drug levels (0.3% *w*/*w*) and different enhancer concentrations showed a significant difference (*p* < 0.0001) in the cumulative amount of drug permeated. A similar observation was also noticed with higher drug levels (0.5 and 0.7% *w*/*w*), where the increase in enhancer concentration increased the transdermal flux. Additionally, these outcomes agreed with earlier research [46]. The possible mechanism for the enhancement in transdermal permeation of treprostinil could be due to its potential to enhance drug solubility in stratum corneum, which could favor the drug partition into the skin and enhance lipid fluidity as well as permeation as described in the literature [33].

Figure 6b compares the permeation profile of the optimized patch with the control (patch prepared without using an enhancer). Two distinct profiles were noticed, with a significantly higher (*p* < 0.001) amount permeating from the optimized patch. The effect of permeation enhancers was much evident here as the cumulative amount permeated (505.59 ± 40.56 µg/cm^2^) from the optimized patch at 24 h was ~6 folds higher than the control (91.85 ± 27.78 µg/cm^2^). The flux (~23.26 µg/cm^2^/h, as well as lag time (~0.06 h) observed were high in the optimized batch as compared to its counterpart (flux; ~3.84 µg/cm^2^/h, and lag time; 1.08 h). The permeability coefficient observed with the optimized patch was also found to be much higher (~23.26 × 10^−3^ cm/h) than its counterpart (~3.83 × 10^−3^ cm/h). Overall, the data here substantiate the possibility of optimized transdermal treprostinil to deliver effectively through the skin. 

#### 3.3.3. Optimized Batch Selected by Point Prediction 

**Figure 7:**
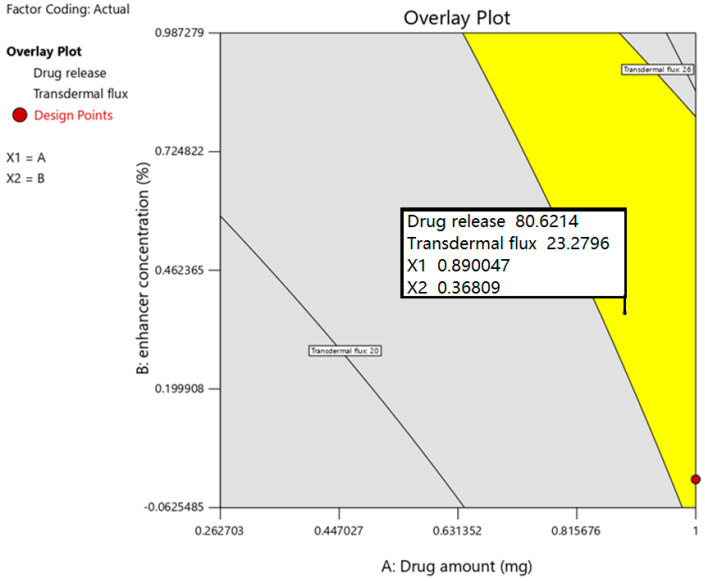
Overlay plot of the optimized drug in the adhesive patch using design space.

Based on DoE batches studies, and statistical and graphical observations, the optimized value of independent variables was selected from the overlay graph using the point prediction method (Design space, Design Expert software), which could provide the responses which were targeted. Marking of the point in the yellow region in the overlay plot depicted in Figure 7 shows estimated values for independent variables and responses. According to point prediction in design space, it shows X_1_—0.890 (0.678% *w*/*w*) and X_2_—0.368 (5.736% *w*/*w*). The estimated and observed values of responses were compared to validate the design model. The results of estimated and observed values of drug release (Y_1_) and transdermal flux (Y_2_) of the optimized formulation are presented in Table 4. In both cases (drug release and transdermal flux), the results show that the values obtained were very near, and the discrepancy between predicted and actual values was found to be minor (<1%). In a nutshell, the data here conclude that the mathematical equation generated is appropriate for estimating the release of the drug and transdermal flux, and hence the model is mathematically valid.

### 3.4. Characterization of Transdermal Patch

#### 3.4.1. Drug Content

Determining the drug content is a crucial quality control tool generally used in pharmaceutical products, including transdermal systems. The average amount of drug determined in the optimized adhesive patch was 97.60 ± 2.15%, demonstrating higher (>95%) treprostinil content. The higher content uniformity noticed here also signifies that the composition in the optimized formulation did not affect the drug level.

#### 3.4.2. Microscopic Observations

**Figure 8:**
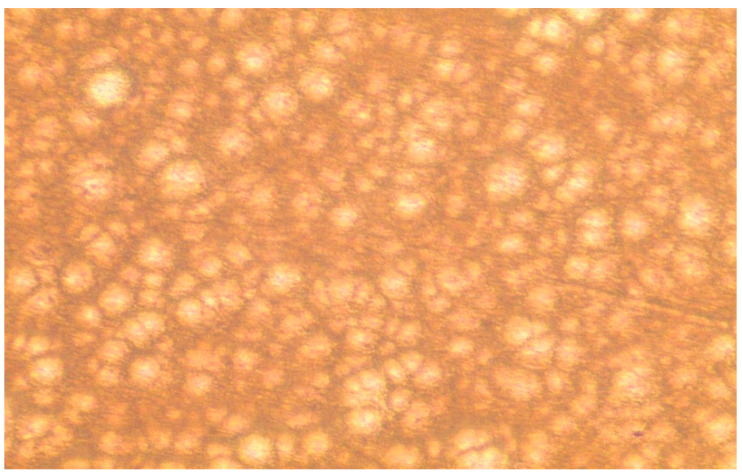
Light microscopy picture of the optimized drug in the adhesive patch.

The surface morphology of the optimized adhesive patch and crystallization of treprostinil were examined using optical and scanning electron microscopy. Figure 8 represents the optical microscopic image of the patch containing treprostinil. It is apparent from the image that the surface of the patch was free from drug crystals, while the drug, adhesive matrix and enhancer are homogeneously distributed. 

**Figure 9:**
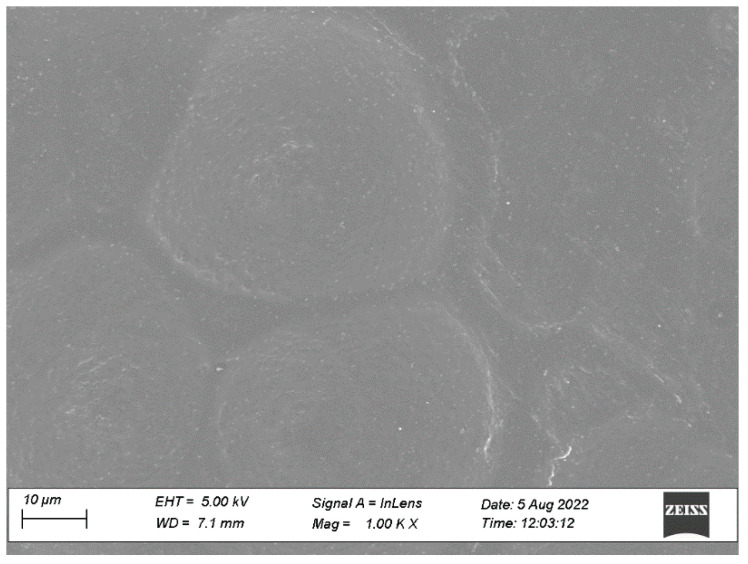
Scanning electron microscopy image of the optimized drug in the adhesive patch.

The microstructure of the patch surface of the prepared transdermal patch could be thoroughly visualized using SEM [47]. Hence, SEM was utilized to examine the topography of the optimized patch. Figure 9 depicts the SEM image of the optimized patch, which signifies the surface is relatively smooth, homogenous, and without any solid particles or cracks. The micrograph also makes it clear that the patch has good integrity. The patches did not show any signs of treprostinil crystals; probably, the drug is in molecular form and embedded in the adhesive. However, a few white spots on the surface could be due to the backscattered light effect, as generally noticed in the SEM images of transdermal patches [47]. Overall, the optimized patch morphology seems suitable for transdermal application. 

#### 3.4.3. FTIR Study

**Figure 10:**
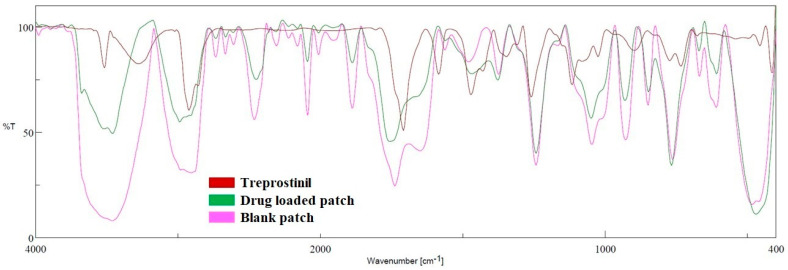
Fourier transforms infrared spectroscopy of treprostinil, blank patch, and optimized drug-loaded patch.

FTIR studies were performed to assess the interaction of the drug with other embodiments used in the patch. In some cases, the presence of specific functional groups in excipients may react with drug compounds and causes their decomposition or make them unstable, which finally affects the stability and bioavailability. Figure 10 shows the FTIR spectra of treprostinil, blank patch, and optimized patch. The specific characteristic peaks of treprostinil observed in Figure 10 and their corresponding functional groups [48] are described in Table 5. In the case of the blank patch, the peaks of adhesive at 3463 cm^−1^ (O-H intermolecular bonded, 2734 cm^−1^ C-H (CH_2_ stretching alkane), 1739 cm^−1^ (C=O stretching—vinyl phenyl ester), 1481 cm^−1^ (C-H bending alkane), 1373 cm^−1^ (O-H bending), 1241 cm^−1^ (-C-O-C), 1045 cm^−1^ (C-O stretching), 929 cm^−1^ (C-O vinyl acetate), and 763 cm^−1^ (C-H bending) were evident [47]. It is evident in Figure 10 that the optimized patch has all major peaks of treprostinil and adhesive. The existence of treprostinil peaks in the optimized patch suggests the drug, as well as the ingredients used to fabricate the patch, are chemically compatible, and the product is likely to have good stability. 

#### 3.4.4. DSC

**Figure 11:**
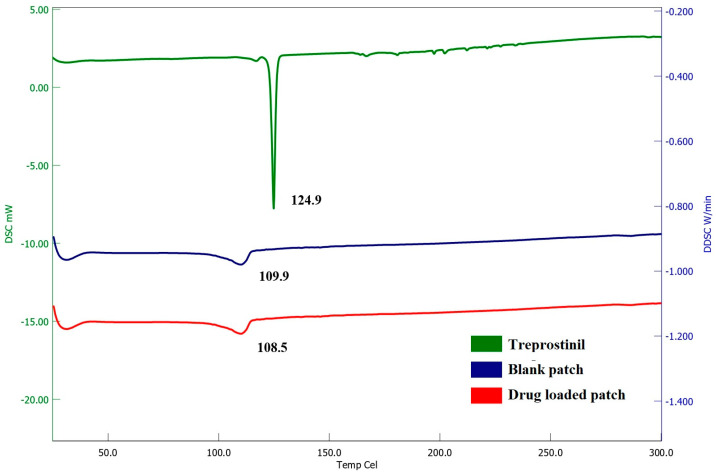
Differential scanning calorimetry thermograms of treprostinil, blank patch, and optimized drug-loaded patch.

The application of DSC enables the quantitative evaluation of all processes that involve the utilization or generation of energy, resulting in either endothermic or exothermic phase transformations. The physical state of treprostinil and potential changes in thermodynamic characteristics within the matrix, along with the thermal stability of the drug/adhesive, could be assessed using the DSC technique. This thermal analysis technique provides information on the changes in melting and solid phase changes as well the change of the treprostinil from crystalline to amorphous. Figure 11 displays the thermograms of treprostinil, blank patch, and optimized drug-loaded adhesive patch. Treprostinil showed a major endothermic peak at 124.9 °C due to the anhydrous crystal form that was used in formulating patches. A minor endothermic peak was visible for both the blank patch and optimized drug-loaded patch at 109.9 °C and 108.5 °C, respectively, probably representing the glass transition temperature of vinyl carboxylic acids monomers in the acrylic adhesive [49]. However, the drug peak was absent in the DSC thermogram of the formulated drug in the adhesive patch, and this could be because of the amorphous form of treprostinil molecules dispersed in the adhesive mixture [50]. The absence of any new endothermic peaks in the prepared patch implies the stability of the drug with the additives used. Overall, the findings of this study show that the drug embedded in the prepared patches exists in an amorphous form.

#### 3.4.5. Coat Weight

Many factors could influence the coat weight of transdermal patches, including the solid content of the adhesive used as well as the patch thickness [51]. By measuring the coat weight at various regions of the laminate, one can ascertain the coating effectiveness of the prepared transdermal patch. To be more accurate, we have excluded the weight of the release liner as well as the backing membrane of the prepared patch. The measured coat weight of the optimized drug in the adhesive patch was 5.3 ± 0.4 mg, demonstrating consistent coat weight across the laminate.

#### 3.4.6. Adhesion Testing 

Peel adhesion and tackiness are commonly used to quantitatively assess the adhesive nature of transdermal patches. The peeling test is a well-established method for analyzing drugs in adhesive transdermal systems that quantifies the force required to remove a patch from an adherend [52]. Furthermore, this test provides patch peeling resistance, which refers to the discomfort experienced during patch removal, and verifies the nonexistence of adhesive residuals on the skin surface [30,53]. The test was performed at a peel angle of 180°, which is thought to be the best for minimizing pain. The observed peel adhesion force of the prepared treprostinil patch was 5198 g (Figure S1), indicating that removing the patch from the skin will not cause pain, as previously reported [54,55]. In addition, the patch was intact and free of major flaws, and no patch residue was found on the stainless steel.

Tack adhesion indicates the bonding of an adhesive patch on the skin surface during a brief period of contact when applied with slight pressure [56]. Hence, the measurement of tackiness is of utmost importance for transdermal patches. In this investigation, the maximum force required to detach the patch after 50 s of adhesion was measured as a tack. The observed tack value of the optimized patch was 442 g (peak load, Figure S2), signifying adequate adhesion as reported earlier [53]. Moreover, the adhesive used in patch preparation (DuroTak 87-2287) has good tackiness, according to the Henkel product selection guide for transdermal pressure-sensitive adhesives.

### 3.5. Skin Irritation

The evaluation of skin irritation of developed drugs in adhesive transdermal patches is essential to ensure the safety of the skin during clinical application. The outcomes of the skin irritancy test show that using a typical skin irritant resulted in a higher main irritancy index (6.23 ± 1.08) compared to using a transdermal placebo patch (0.79 ± 0.51) and using an optimized drug-loaded patch (1.03 ± 0.64). Furthermore, neither the placebo nor the drug-loaded adhesive patches caused any discernible edema on the rat’s skin. These findings imply that the adhesive patch medication and the placebo are not skin irritants, as reported earlier [31].

### 3.6. In Vivo Study

**Figure 12:**
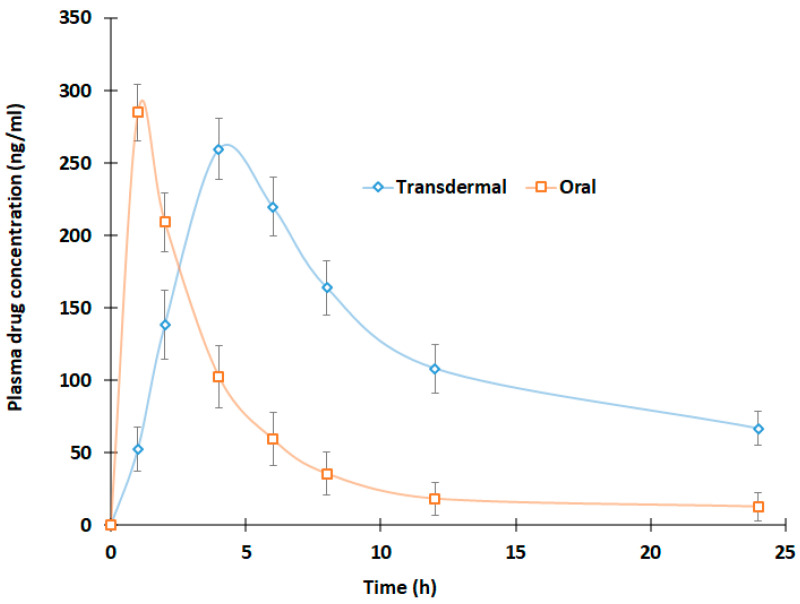
Plasma concentration-time plot of treprostinil from optimized patch and control (oral) (*n* = 6).

The evaluation of the optimized patch in rats aimed to comprehend the pharmacokinetics of treprostinil in the patch formulation. The pharmacokinetics profile of treprostinil following transdermal delivery was compared with oral administration of the same dose (Figure 12). The calculated C_max_, T_max_, and AUC_0–α_ values are depicted in Table 6. The plasma profiles in Figure 12 indicate two noticeably different plasma treprostinil curves when the administration route was changed. In the case of transdermal therapy, the plasma treprostinil level was relatively low in the first hour (52.55 ± 15.50 ng/mL) as compared to the oral counterpart (284.87 ± 19.38 ng/mL). The drug concentration in transdermal therapy further increased steadily in a time-dependent manner and reached C_max_ (259.75 ± 21.22 ng/mL) in 4 h (T_max_). Indeed, the C_max_ values of oral and transdermal therapy were found to be comparable (*p* = 0. 0579). After the C_max_, the drug amount decreases in both routes, while the elimination was rapid in the case of oral (Figure 12). The higher rate of treprostinil absorption from transdermal therapy was evidenced by greater AUC_0-α_ (*p* < 0.0001, Table 5) as compared with oral administration of treprostinil suspension. This finding demonstrated that treprostinil drug in the adhesive patch has a much higher relative bioavailability (237%) than the oral treprostinil suspension without appreciable changes in peak exposure. The overall results here suggest that the drug in an adhesive patch of treprostinil is capable of enhancing the drug’s bioavailability, demonstrating the prospect of transdermal delivery in treating PAH.

## 4. Conclusions

A systematic study was conducted to formulate a treprostinil transdermal patch by evaluating various pharmaceutical properties. Formulation components were screened in the initial phase of the investigation, and optimization of drug amount and chemical enhancer was performed using DOE. The optimization data suggests both independent variables influence the responses (drug release from the patches and transdermal flux) significantly, but not to the same extent. The characterization of the optimized patch indicates the developed patch possesses all properties suitable for in vivo investigation. The higher treprostinil absorption in rats by transdermal therapy was evidenced by greater AUC_0-α_ (*p* < 0.0001) and relative bioavailability (237%) as compared with peroral. It should be emphasized that the results observed here are in rats and can be valuable preliminary data for formulation development, and the extrapolation to the human should be made carefully. Therefore, it is crucial to conduct further studies and clinical trials in human subjects to validate the findings and optimize the drug formulation for human use in the management of PAH.

## Figures and Tables

**Table 1:** Level and actual coded values used for optimization.

Factors, Response	Level Used, Actual Coded		
	Low (−1)	Medium (0)	High (+1)
Independent variables			
X_1_ = Drug amount (%, *w*/*w*)	0.3	0.5	0.7
X_2_ = Enhancer concentration (%, *w*/*w*)	3	5	7
Dependent variables	Targets		
Y_1_ = Drug release (%)	Maximum		
Y_2_ = Transdermal flux (µg/cm^2^/h)	Maximum		

**Table 2:** Concentrations of various factors are used for optimization.

Batch	Factor 1	Factor 2
	X_1_: Drug Amount (%)	X_2_: Enhancer Concentration (%)
F1	0	−1
F2	−1	−1
F3	1	0
F4	−1	0
F5	1	1
F6	1	−1
F7	−1	1
F8	0	0
F9	0	1

**Table 3:** Factors used and their response during optimization.

Batch Number	Factor 1	Factor 2	Response 1	Response 2
	X_1_: Drug Amount (%, *w*/*w*)	X_2_: Enhancer Concentration (%, *w*/*w*)	Y_1_: Drug Release (%)	Y_2_: Transdermal Flux (µg/cm^2^/h)
F1	0.5	3	60.23	14.54
F2	0.3	3	45.23	10.34
F3	0.7	5	80.12	22.96
F4	0.3	5	50.11	12.12
F5	0.7	7	85.99	26.98
F6	0.7	3	76.91	19.45
F7	0.3	7	55.41	15.67
F8	0.5	5	65.05	16.28
F9	0.5	7	70.24	19.98

**Table 4:** Estimated and observed values of dependent variables of the optimized formulation.

Y_1_ (Drug Release) %		Y_2_ (Transdermal Flux) µg/cm^2^/h	
Estimated	Observed	Estimated	Observed
80.621	81.341 ± 4.93	23.279	23.259 ± 3.54

**Table 5:** Absorption peaks of treprostinil observed in FTIR and its corresponding functional groups.

Wave Numbers (cm^−1^)	Functional Groups
3517	O-H stretching-alcohol
3278	O-H stretching-carboxylic acid
2923	C-H stretching
1707	C=O stretching
1585	C=C stretching
1469	C-H bending
1346	O-H bending
1261	C-O stretching
1114	C-O stretching—secondary alcohol
1025, 898, 732	C=C bending

**Table 6:** In vivo parameters of treprostinil in plasma after oral and transdermal delivery in rats.

Parameters	Treprostinil Suspension (Mean ± SD)	Treprostinil Transdermal Patch (Mean ± SD)
C_max_ (ng/mL)	284.87 ± 19.44	259.75 ± 21.28
T_max_ (h)	1	4
AUC_0-α_ (ng.h/mL)	1351.49 ± 106.43	3977.11 ± 287.65 *
Relative bioavailability (%)	100	237

* *p* < 0.0001 vs. treprostinil suspension.

## Data Availability

The data presented in this study are contained within the article.

## Supplementary Materials

The following supporting information can be downloaded at: https://www.mdpi.com/article/10.3390/pharmaceutics15041226/s1, Table S1. Composition of preliminary trial batches (F1–F20) of transdermal patch; Table S2. Flux values observed in preliminary trial batches (F1–F20) of transdermal patch; Table S3. ANOVA for Quadratic model for drug release; Table S4. ANOVA for quadratic model for transdermal flux; Table S5. Model fitting for optimized Treprostinil patch; Figure S1. Load versus time curve of the peeling force of optimized patch; Figure S2. Load versus time curve of the tackiness of optimized patch.
