# Supplementary material for: Design, Development, and Evaluation of Treprostinil Embedded Adhesive Transdermal Patch
Source: Pharmaceutics. 2023 Apr 12;15(4):1226. doi: 10.3390/pharmaceutics15041226 (PMC10146406; doi:10.3390/pharmaceutics15041226)
Supplement: Supplementary file 1 [file pharmaceutics-15-01226-s001.zip › pharmaceutics-2329948-supplementary.pdf]

pharmaceutics

MDPI

Article

Design, Development, and Evaluation of Treprostinil Embedded Adhesive Transdermal Patch

Ibrahim Alissa [1], Anroop B. Nair [1,*], Bandar Aldhubiab [1], Hiral Shah [2], Jigar Shah [3], Vivek Mewada [3], Rashed M. Almuqbil [1] and Shery Jacob [4]

1 Department of Pharmaceutical Sciences, College of Clinical Pharmacy, King Faisal University, Al-Ahsa 31982, Saudi Arabia; 221401354@student.kfu.edu.sa (I.A.); baldhubiab@kfu.edu.sa (B.A.); ralmuqbil@kfu.edu.sa (R.M.A.)
2 Department of Pharmaceutics, Arihant School of Pharmacy & BRI, Adalaj, Gandhinagar 382421, India; vyashiral@yahoo.co.in
3 Department of Pharmaceutics, Institute of Pharmacy, Nirma University, Ahmedabad 382481, India; jigsh12@gmail.com (J.S.); vmewada91@gmail.com (V.M.)
4 Department of Pharmaceutical Sciences, College of Pharmacy, Gulf Medical University, Ajman 4184, United Arab Emirates; sheryjacob6876@gmail.com
* Correspondence: anair@kfu.edu.sa

Table S1. Composition of preliminary trial batches (F1-F20) of transdermal patch.

Formula Code	Ingredients	
	Treprostinil (% w/w)	Propylene Glycol (% w/w)
F1	0.1	5
F2	0.2	5
F3	0.3	5
F4	0.4	5
F5	0.5	5
F6	0.6	5
F7	0.7	5
F8	0.8	5
F9	0.9	5
F10	1	5
F11	0.5	1
F12	0.5	2
F13	0.5	3
F14	0.5	4
F15	0.5	5
F16	0.5	6
F17	0.5	7
F18	0.5	8
F19	0.5	9
F20	0.5	10

Table S2. Flux values observed in preliminary trial batches (F1-F20) of transdermal patch.

Formula Code	Transdermal Flux (μg/cm²/h)
F1	05.45 ± 2.45
F2	09.03 ± 8.65
F3	13.65 ± 4.32
F4	19.37 ± 7.34
F5	23.04 ± 3.56
F6	23.82 ± 2.98
F7	25.73 ± 1.89
F8	24.88 ± 3.44
F9	24.22 ± 6.03
F10	25.75 ± 5.17
F11	08.63 ± 3.66
F12	16.09 ± 2.84
F13	20.83 ± 1.09
F14	21.12 ± 6.21
F15	23.36 ± 4.69
F16	24.89 ± 5.37
F17	24.91 ± 6.41
F18	24.44 ± 8.03
F19	23.15 ± 7.31
F20	23.95 ± 4.82

Table S3. ANOVA for Quadratic model for drug release.

Source	Sum of Squares	df	Mean Square	F-value	p-value	
Model	1563.13	5	312.63	1516.70	< 0.0001	significant
A-Drug amount	1418.96	1	1418.96	6884.07	< 0.0001	
B-enhancer concentration	142.79	1	142.79	692.74	0.0001	
AB	0.3025	1	0.3025	1.47	0.3124	
A^2	0.4141	1	0.4141	2.01	0.2514	
B^2	0.6613	1	0.6613	3.21	0.1712	
Residual	0.6184	3	0.2061			
Cor Total	1563.74	8				

Table S4. ANOVA for quadratic model for transdermal flux.

Source	Sum of Squares	df	Mean Square	F-value	p-value	
**Model**	222.84	5	44.57	250.51	0.0004	significant
A-Drug amount	162.86	1	162.86	915.46	< 0.0001	
B-enhancer concentration	55.82	1	55.82	313.74	0.0004	
AB	1.21	1	1.21	6.80	0.0798	
A^2	1.95	1	1.95	10.94	0.0455	
B^2	0.9988	1	0.9988	5.61	0.0986	
**Residual**	0.5337	3	0.1779			
**Cor Total**	223.37	8				

Table S5. Model fitting for optimized Treprostinil patch.

Model Name	Multiple R	R Square	X variable	Slope	SSR	Fischer Ratio
**Zero Order**	0.9660	0.9332	6.4993	12.7241	469.0102	67.0015
**First Order**	0.9981	0.9962	-0.0594	1.9703	63.8188	9.1170
**Higuchi**	0.9977	0.9954	24.8690	-2.9082	32.5762	4.6537
**Korsmeyer - Peppas**	0.9957	0.9913	0.6089	-0.7131	68.8329	9.8333
**Weibull Model**	0.9994	0.9989	0.8130	-0.6631	7.7355	1.1051
**Hixson - Crowell**	0.9921	0.9843	0.1621	0.1544	155.3547	22.1935

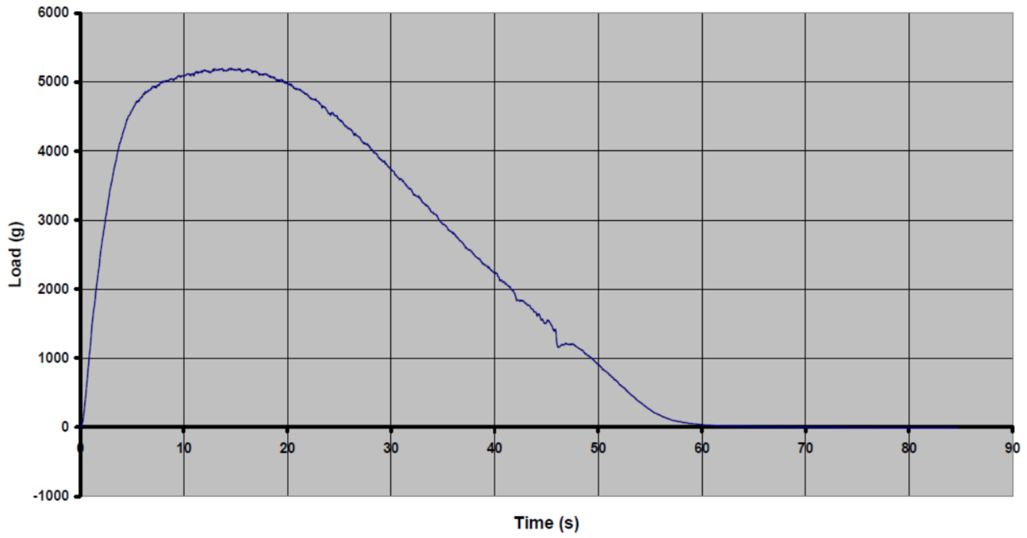

Figure S1. Load versus time curve of peeling force of optimized patch.

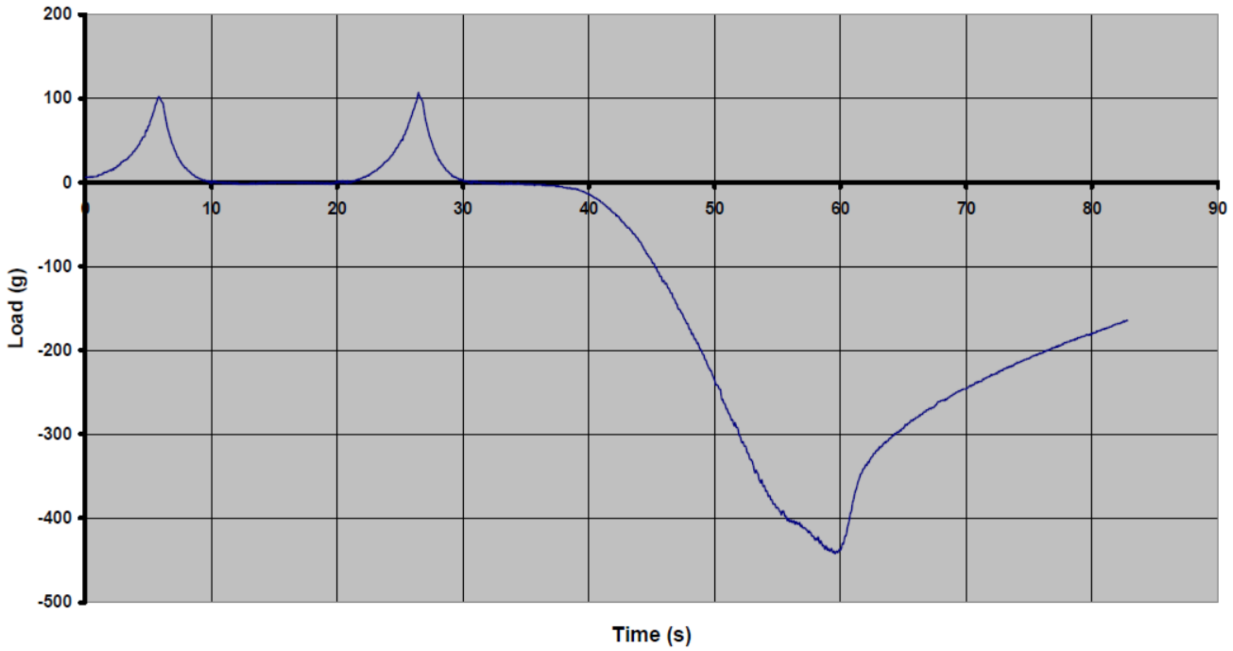

Figure S2. Load versus time curve of tackiness of optimized patch.
